# 肺静脉跨叶现象及其在肺叶切除术中的临床意义

**DOI:** 10.3779/j.issn.1009-3419.2021.104.01

**Published:** 2021-02-20

**Authors:** 文正 徐, 志华 李, 志成 何, 晶 许, 卫兵 吴, 亮 陈

**Affiliations:** 210029 南京，南京医科大学第一附属医院（江苏省人民医院）胸外科 Department of Thoracic Surgery, The First Affiliated Hospital of Nanjing Medical University, Nanjing 210029, China

**Keywords:** 肺结节, 肺跨叶静脉, 肺叶切除术, 3D-CTBA, 解剖变异, Pulmonary nodules, Translobar veins, Lobectomy, 3D-CTBA, Anatomical variation

## Abstract

**背景与目的:**

肺叶切除术是早期肺癌的主要治疗方式，然而肺部解剖变异繁多，尤其是部分肺静脉会脱离正常解剖位置，甚至跨越肺叶边界进入相邻肺叶，这增加了手术的难度和风险。本研究旨在全面分析肺跨叶静脉的类型和发生频率，并进一步探究其在肺叶切除术中的临床意义。

**方法:**

纳入南京医科大学第一附属医院2018年12月-2019年11月期间行肺部手术的患者916例，筛选出术前行胸部增强计算机断层扫描（computed tomography, CT）检查的病例310例，应用这些患者的胸部增强CT和三维CT支气管血管成像（three-dimensional computed tomography bronchography and angiography, 3D-CTBA）对跨叶静脉的类型及频率进行分析。进一步从916例手术病例中筛选出行肺叶切除术且手术区域涉及到跨叶静脉的病例共48例（切断组36例，保留组12例），通过手术录像观察两种不同的处理方式对余肺叶的影响。

**结果:**

共发现26种肺跨叶静脉模式，总发生率为82.26%，右肺远大于左肺（80.65% *vs* 11.94%）。右肺中主要类型（频率 > 5%）包括：中间支气管后方汇入下肺静脉的VX2（5.48%）、汇集上、下叶静脉属支的叶间静脉V^3^b（58.39%）、水平裂内汇入V^2^（13.23%）或V^3^（12.58%）的VX^4^、中叶支气管下方汇入左心房的VX^4^（8.71%）或VX^5^（7.42%）、肺裂内汇入V^2^的VX^6^（29.68%）；左肺中主要类型为整支上肺静脉汇入下肺静脉形成共干的左肺静脉（9.36%），其中共干长度 > 1 cm的占4.84%。观察手术录像发现：与保留组相比，切断组中患者其余肺叶的血液循环功能受到影响（表现为受影响区域停止通气后长时间无法萎陷），术后咯血（13.89% *vs* 0.00%）和肺漏气（19.44% *vs* 8.33%）发生率增加，术后住院时间[(4.72±1.86) d *vs* (3.92±1.62) d]较长，术后3 d引流量[(705.42±265.02) mL *vs* (604.92±229.64) mL]较多，但差异无统计学意义（*P* > 0.05）。

**结论:**

肺静脉跨叶现象种类繁多，且部分类型发生率较高。然而大部分跨叶静脉在手术中被忽视，这可能对手术安全和患者术后恢复产生不利影响。

## Abstract (en)

**Background and objective:**

Lobectomy is the main treatment strategy for early lung cancer. However, there are many anatomical variations in the lungs, especially some pulmonary veins will deviate from the normal position, and even cross the border of lob to enter the adjacent lobes, which increases the difficulty and risk of lobectomy. This study aims to analyze the variation types and frequency of pulmonary translobar veins, and further investigate its clinical significance in lobectomy.

**Methods:**

This study retrospectively included 916 patients who underwent lung resection in our center from December 2018 to November 2019, and selected 310 patients who underwent enhanced chest computed tomography (CT) examination before surgery. Enhanced chest CT and three-dimensional computed tomography bronchography and angiography (3D-CTBA) of these patients were applied to analyze the types and frequency of translobar veins. Further, 48 cases of lobectomy whose operation areas involved translobar veins were further screened out of 916 surgical cases (Among them, the translobar veins of 36 subjects were cut off and the other 12 patients were reserved). The effect of two different treatments on remaining lung was observed by surgical video.

**Results:**

A total of 26 translobar veins patterns were identified with an overall incidence of 82.26%, much greater in the right than in the left lung (80.65% *vs* 11.94%). The major types (frequency > 5%) in the right lung include: the VX2 (5.48%) that flows into the inferior pulmonary vein behind the intermediate bronchus, the V^3^b (58.39%) that converges the venous branches of the upper and middle lobe, the VX^4^ that flows into the V^2^ (13.23%) or V^3^ (12.58%) in the horizontal fissure, the VX^4^ (8.71%) or VX^5^ (7.42%) that flows into the left atrium beneath the middle lobe bronchus, and the VX^6^ that flows into V^2^ in the Oblique fissure. The major types in the left lung include the common trunk (9.36%) of the superior and inferior pulmonary vein, of which 4.84% are greater than 1 cm in length. Compared to the preserved group, circulatory function of the remaining lung was impaired in the severed group, with the impaired area failing to collapse long after ventilation is stopped, the incidence of postoperative hemoptysis (13.89% *vs* 0.00%) and pulmonary air leakage (19.44% *vs* 8.33%) increased, the postoperative hospital stay [(4.72±1.86) d *vs* (3.92±1.62) d] was longer, and the total drainage during 3 days after operation [(705.42±265.02) mL *vs* (604.92±229.64) mL] was more, but the difference is not statistically significant.

**Conclusion:**

There are a variety of types of translobar pulmonary veins and some of them have a high incidence; However, most of the translobar veins were neglected in surgery, which could adversely affect the safety of surgery and the recovery of patients after surgery.

肺癌的发病率和死亡率居各种恶性肿瘤之首^[1]^，肺叶切除术是早期肺癌的主要治疗手段^[2]^。成功的手术不仅需要安全地切除病变肺叶，还不应影响到保留肺叶的生理功能。然而肺部解剖变异繁多，尤其是部分肺静脉会脱离正常解剖位置，甚至跨越肺叶边界进入相邻肺叶，这增加了手术的难度和风险，导致术中意外出血以及在切除病变肺叶的同时保留肺叶的生理功能受到影响。

关于肺静脉的跨叶现象，既往有许多学者做过相关的描述。早些年Yamashita^[3]^、刘正津等^[4]^在标本解剖过程中发现，每一例相邻肺叶肺实质融合的标本内都可见小静脉跨叶分布，但他们并未针对这些跨叶静脉的来源进行详细分析。随后Otsuji等^[5]^通过分析胸部计算机断层扫描（computed tomography, CT）发现，超过95%不完全发育的叶裂内可见支气管、血管跨越两个相邻的肺叶，并进一步对其来源进行了分析，但由于样本数较少，一些特殊的跨叶静脉类型没有包含在内。近些年来，一部分学者开始利用三维CT支气管血管成像（three-dimensional computed tomography bronchography and angiography, 3D-CTBA）技术研究肺静脉的变异现象^[6-8]^。然而，既往这些研究都没有针对肺静脉跨叶现象进行全面而深入的分析，而且研究内容都集中在现象描述层面上，没有进一步详细分析跨叶静脉对于肺叶切除术的影响。

本研究即从肺叶切除手术出发，将这些解剖位置特殊且对手术操作有直接影响的静脉总结为跨叶静脉，同时结合增强CT、3D-CTBA和手术录像对跨叶静脉的类型及频率进行系统性分析，并进一步阐述跨叶静脉的临床意义和处理策略。

## 1 材料与方法

### 1.1 临床资料

回顾性分析南京医科大学第一附属医院胸外科同一治疗组2018年12月-2019年11月期间连续进行胸腔镜肺部手术的病例916例，其中术前进行胸部增强CT检查的病例324例。排除14例，最终纳入病例总数310例，患者基本临床信息见表 1。排除标准：①CT图像模糊且3D-CTBA不能清晰地显示支气管、血管亚段分支；②既往有肺部手术史；③病历资料不全。

### 1.2 增强CT和三维重建方法

CT的机型是西门子第一代双源CT（Somatom Definition），对比剂是碘普罗胺370。首先，用团注试验法来获得时间-密度曲线，用以设定扫描开始的时刻和对比剂的用量。然后将计算的对比剂用量以5 mL/s的注射速度机械注入，随后以相同流速追加20 mL生理盐水。然后在特定的时间进行扫描，扫描时准直器厚度为0.6 mm，重建层厚为1 mm，层间距为1 mm，重建的卷积核（kernel）为软组织算法（B30）。即可在一次扫描中显示肺动脉、肺静脉及其分支，并且肺动脉和肺静脉显示有密度差异。将CT图像导入我中心与东软医疗、东北大学共同研发的三维可视化软件DeepInsight进行支气管、血管重建。

### 1.3 跨叶静脉的定义

结合既往文献^[5]^，本研究将跨叶静脉分为以下三类：①肺静脉跨越叶间裂进入相邻肺叶，或直接汇入左心房；②叶间静脉走行于叶裂内汇集相邻两叶的属支；③肺静脉汇入体循环静脉系统。结合增强CT图像和3D-CTBA图像观察是否存在跨叶静脉。肺段结构命名采用陈亮等描述的支气管、血管命名法^[9]^，把正常肺段或亚段所处位置的来源于临近肺段或亚段的静脉命名为VX^[10]^。

### 1.4 跨叶静脉影响肺叶切除术的验证方法

基于上述肺跨叶静脉划分方法，本研究进一步回顾此916例手术患者中行肺叶切除术的患者共203例，筛选出手术区域涉及到跨叶静脉的病例48例，根据术中跨叶静脉的处理方式分为保留组和切断组，回顾手术录像及影像资料，观察不同处理方式对保留肺叶的影响，并比较两组患者围术期资料。其中术后咯血定义为术后痰中带血≥3 d，肺漏气定义为术后漏气≥3 d，低白蛋白血症定义为血清白蛋白低于35 g/L。

### 1.5 统计学方法

采用SPSS 25.0统计学软件进行统计学分析，计量资料结果用均数±标准差（Mean±SD）表示，采用*t*检验；计数资料采用*χ*^2^检验或*Fisher*精确检验。*P* < 0.05（双侧检验）为差异有统计学意义。

## 2 结果

### 2.1 临床资料

筛选出310例患者进行跨叶静脉分析，其中男性107例，女性203例，平均年龄（53.47±11.46）岁；肺结节平均直径（10.57±7.79）mm；术后病理中47例原位腺癌（adenocarcinoma *in situ*, AIS），88例微浸润腺癌（minimally invasive adenocarcinoma, MIA），146例浸润性腺癌（invasive adenocarcinoma, IAC），1例鳞癌，28例良性病变。研究对象具体基线及临床病理信息见表 1。

**1:** 研究对象基线及临床病理特征 Baseline and clinicopathological characteristics of subjects

General data	Data
Gender	
Male	107 (34.52%)
Female	203 (65.48%)
Age (Mean±SD, yr)	53.47±11.46
Diameter of nodules (mm)	10.57±7.79
Histologic diagnosis	
Adenocarcinoma	281 (90.65%)
Squamous cell carcinoma	1 (0.32%)
Benign lesion	28 (9.03%)

### 2.2 跨叶静脉的类型和发生率

肺跨叶静脉的发生率为82.26%（表 2），右肺远大于左肺（右肺：80.65%；左肺：11.94%；其中有10.33%患者右肺与左肺同时存在跨叶静脉），主要有以下3种。

**2:** 肺跨叶静脉的类型及各类型发生频率 Types of pulmonary translobar veins and frequency of each type

Translobar vein	Location	Inflow site	Number	Frequency
Right lung			250	80.65%
V^2^	Behind the BI	SPV	4	1.29%
	Behind the BI	IPV	17	5.48%
	Behind the BI	LA	7	2.26%
	Between B^6^ and CBB	V^6^	1	0.32%
V^3^b	Horizontal fissure	SPV	181	58.39%
V^4^	Horizontal fissure	V^2^	41	13.23%
	Horizontal fissure	V^3^	39	12.58%
	Below the MLB	IPV	11	3.55%
	Front of the hilum	LA	27	8.71%
	Outside of CBA	V^6^	1	0.32%
V^5^	Front of the hilum	V^3^	3	0.97%
	Below the MLB	IPV	2	0.65%
	Front of the hilum	LA	23	7.42%
V^6^	Behind the BI or CBB	SPV	5	1.61%
	Behind the CBB	LA	5	1.61%
	Oblique fissure	V^2^	92	29.68%
V^8^	Above the IPV	LA	1	0.32%
Left lung			37	11.94%
V^1+2^	Behind the LLB	IPV	1	0.32%
	Between B^6^ and CBB	IPV	2	0.65%
Inter.V	Oblique fissure	SPV	3	0.97%
V^4^	Below the ULB	IPV	4	1.29%
	Front of the hilum	LA	2	0.65%
V^5^	Below the ULB	IPV	4	1.29%
	Front of the hilum	LA	2	0.65%
SPV	Lower front of the hilum	IPV	29	9.36%
	Upper front of the hilum	LIV	1	0.32%
Whole lung			255	82.26%
Inter.V: interlobar vein; BI: bronchus intermedius; CBB: common basal bronchus; MLB: middle lob bronchus; CBA: common basal artery; LLB: lower lob bronchus; ULB: upper lob bronchus; SPV: superior pulmonary vein; IPV: inferior pulmonary vein; LA: left atrium; LIV: left innominate vein.				

#### 2.2.1 上叶或下叶的引流静脉分别异常汇入下肺静脉或上肺静脉，或直接汇入左心房

右肺上叶VX^2^从中间支气管后方汇入上肺静脉（4例，1.29%，图 1A）、下肺静脉（17例，5.48%，图 1B）、左心房（7例，2.26%，图 1C），另外有1例VX^2^从B^6^与基底段支气管夹角处下行汇入V^6^（0.32%，图 1D）；左肺上叶VX^1+2^从中间支气管后方汇入下肺静脉仅1例（0.32%），另有2例从B^6^与基底段支气管夹角处下行汇入V^6^（0.65%）。右肺下叶VX^6^在中间支气管或基底段支气管后方汇入上肺静脉（5例，1.61%，图 1A）、左心房（5例，1.61%，图 1M）、在肺裂内汇入V^2^（92例，29.68%，图 1F）。左侧未见上述种类的VX^6^变异，但是发现29例（9.36%，图 1O）上肺静脉向下方走行，在肺门前下方汇入下肺静脉，形成共干的左肺静脉汇入左心房，共干长度平均1.06 cm，有15例（4.84%）超过1 cm。另外发现一种罕见变异：1例部分肺静脉异位引流（0.32%，图 1P），为左上肺静脉垂直向上汇入左无名静脉，此例左肺上叶无其余引流静脉。

**1:**
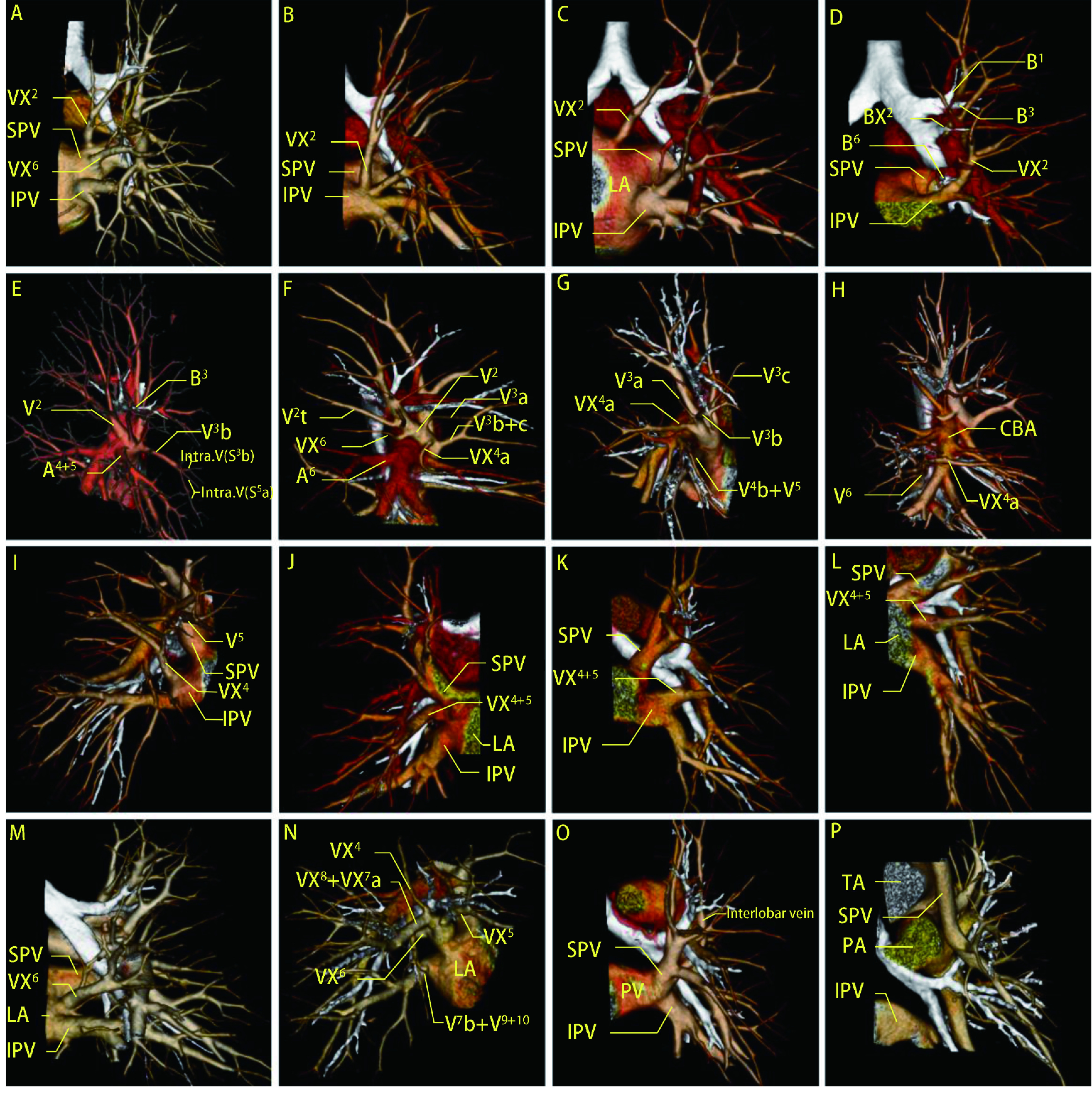
肺跨叶静脉主要类型三维重建图。A-C：右肺VX^2^从BI后方依次汇入SPV、IPV和LA；D：右肺VX^2^从B^6^与CBB夹角处下行汇入V^6^；E：叶间静脉V^3^b；F-H：右肺VX^4^在水平裂内依次汇入V^2^和V^3^，在CBA外侧汇入IPV；I-L：右、左肺VX^4+5^在中叶或舌段支气管下方汇入IPV或LA；M：右肺VX^6^在CBB后方汇入LA；N：右肺VX^4+5^与VX^6^、VX^7^a、VX^8^共干单独汇入LA；O：左肺SPV向下汇入IPV，共干长度为2.14 cm；P：左肺SPV汇入左无名静脉。RUL：右肺上叶；RML：右肺中叶；RLL：右肺下叶；PV：肺静脉；Asc.A^2^：后段动脉后升支。 3D-CTBA of the main types of pulmonary translobar veins. A-C: The right VX^2^ flows into the SPV, IPV and LA sequentially from posterior to the BI; D: The right VX^2^ flows into V^6^ at the angle between B^6^ and CBB; E: Interlobar vein V^3^b; F-H: The right VX^4^ flows into V^2^ and V^3^ sequentially in the horizontal fissure and into IPV laterally in the CBA; I-L: The right and left VX^4+5^ flow into IPV or LA below the MLB or ULB; M: The right VX^6^ flows into LA posterior to CBB; N: The common trunk of right VX^4+5^, VX^6^, VX^7^a and VX^8^ flows into LA separately; O: The left SPV flows downward into the IPV, with a common trunk length of 2.14 cm; P: The left SPV flows into the LIV; RUL: right upper lob; RML: right middle lobe; RLL: right lower lobe; PV: pulmonary vein; Asc.A^2^: ascending A^2^. 3D-CTBA: three-dimensional computed tomo-graphy bronchography and angiography.

#### 2.2.2 V^4+5^部分分支汇入V^2^、V^3^、下肺静脉或左心房

右肺VX^4^可汇入V^2^（41例，13.23%，图 1F）、V^3^（39例，12.58%，图 1G）、下肺静脉（11例，3.55%，图 1I，图 3E）、左心房（27例，8.71%，图 1J），见1例（0.3%，图 1H）右肺VX^4^从基底段动脉外侧向后汇入V^6^，既往文献中未见过此类变异的报道，在汇入左心房的27例中有1例（0.32%）VX^4^与VX^6^共干单独汇入左心房，有1例（0.32%，图 1N）VX^4+5^与VX^6^、VX^7^a、VX^8^共干单独汇入左心房，既往文献中未见过上述两种变异的报道；右肺VX^5^可汇入V^3^（3例，0.97%）、下肺静脉（2例，0.65%）、左心房（23例，7.42%，图 1J）。左肺稍有不同，未见舌段静脉分裂成数支，所见均为舌段静脉整支汇入下肺静脉（4例，1.29%，图 1K）、左心房（2例，0.65%，图 1L）。

**2:**
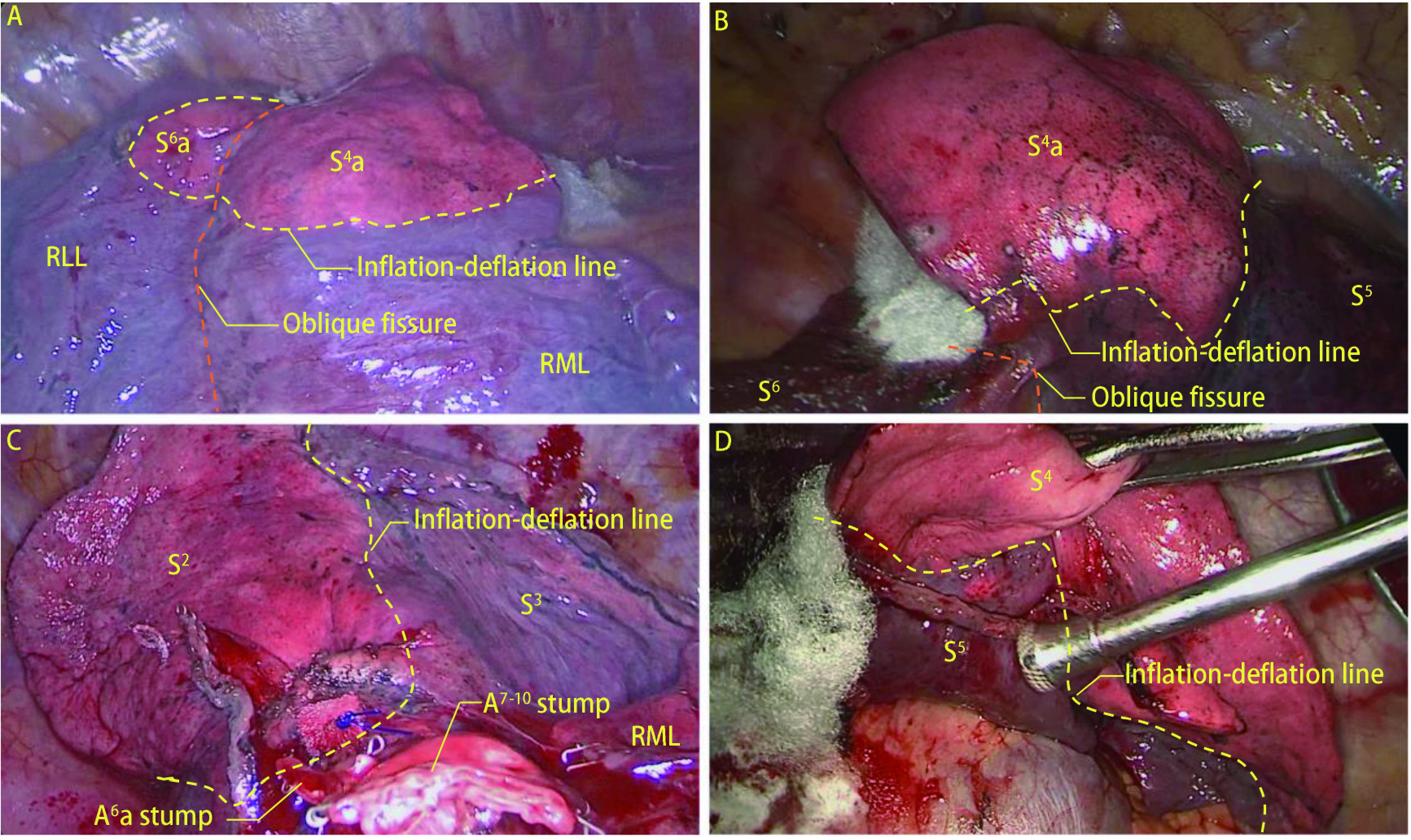
术中切断肺跨叶静脉对余肺萎陷的影响。A-B：右肺上叶切除术中切断汇入V^2^的VX^4^及VX^6^，造成部分S^4^和S^6^血液回流障碍，形成膨胀萎陷界限；C：右肺下叶切除术中切断汇入IPV的VX^2^，形成的膨胀萎陷界限；D：右肺下叶切除术中切断汇入IPV的VX^4^，形成膨胀萎陷界限。 The effect of severing pulmonary translobar veins during operation on the collapse of the remaining lung. A-B: During the right upper lobectomy, the VX^4^ and VX^6^ that flowed into IPV was severed, resulting in the obstruction of partial S^4^ and S^6^ blood flow and forming an inflation-deflation line; C: During the right lower lobectomy, the VX^2^ that flowed into IPV was severed, forming an inflation-deflation line; D: During the right lobectomy, the VX^4^ flowing into IPV were severed, forming an inflation-deflation line.

**3:**
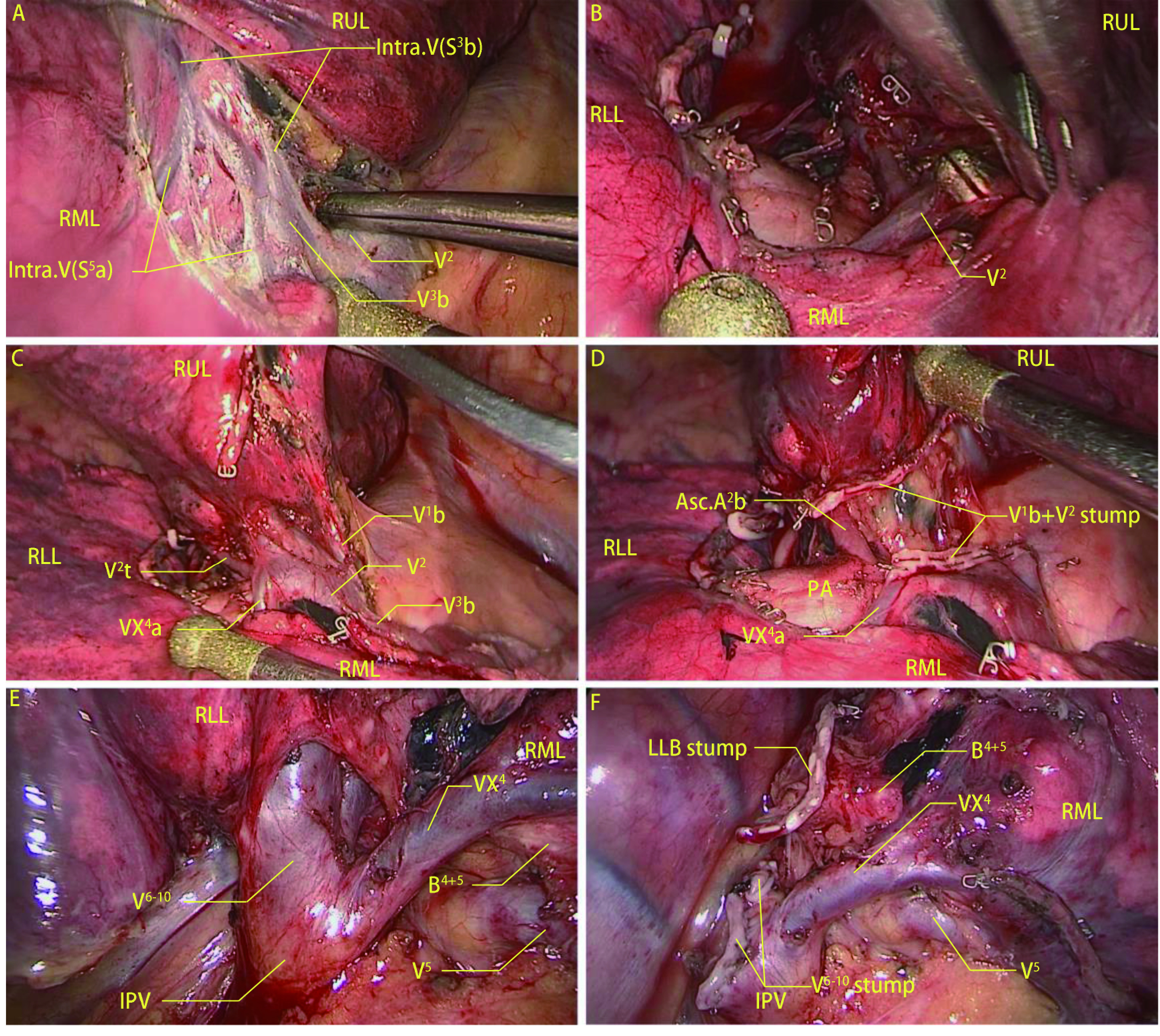
两种典型跨叶肺静脉的保留方法。A-D：右肺上叶切除术中保留汇入V^2^的VX^4^和叶间静脉V^3^b；E-F：右肺下叶切除术中保留汇入IPV的VX^4^。 The methods of preserving two typical pulmonary translobar veins. A-D: The processes that preserve the VX^4^ flowing into V^2^ and interlobar vein V^3^b during the right upper lobectomy; E, F: The VX^4^ flowing into IPV was preserved during the right lower lobectomy.

#### 2.2.3 叶间静脉走行于叶裂内汇集相邻两叶的属支

在水平裂发育不完全时，V^3^b通常作为上叶与中叶的叶间静脉走行于水平裂，共有181例（58.4%，图 1E，图 3A）。另见3例左肺斜裂内叶间静脉（0.97%，图 1O）。

### 2.3 跨叶静脉对肺叶切除术的影响

筛选出48例患者进行外科验证（因汇入V^2^的VX^6^分支过细故未纳入其中），其中切断组36例，保留组12例。切断组中包含34例右肺上叶切除手术中切断叶间静脉V^3^b或汇入V^2^或V^3^的中叶静脉，1例右肺下叶切除术中切断VX^2^和VX^4^，1例左肺下叶切除术中切断VX^4+5^部分分支；保留组中相对应的分别有9例、2例、1例。标本移除后常规进行充气膨胀验漏，而后行单肺通气，观察到切断组中有32例患者余肺受到影响，右肺上叶切除术中切断跨叶中叶静脉造成部分中叶肺组织10 min内无法完全塌陷（图 2A、图 2B）。右肺下叶切除术中切断VX^2^造成部分上叶肺组织无法完全塌陷（图 2C），切断跨叶中叶静脉造成部分中叶肺组织无法完全塌陷（图 2D）。保留组中所有患者余肺均可完全塌陷，右肺上叶切除术中在V^3^b和V^2^上方建立隧道打开水平裂，仅离断上肺静脉上叶侧属支，VX^4^a和叶间静脉V^3^b得以保留（图 3A-图 3D），中叶肺组织可以在10 min内完全塌陷。右肺下叶切除术中将下肺静脉向远端游离即可保留跨叶的中叶静脉（图 3E、图 3F），中叶肺组织也可以完全塌陷。切断组相比于保留组，术后咯血（13.89% *vs* 0.00%, *P*=0.312）、肺漏气（19.44% *vs* 8.33%, *P*=0.659）发生率增加，术后住院时间[(4.72±1.86) d *vs* (3.92±1.62) d]较长，术后3日引流量[(705.42±265.02) mL *vs* (604.92±229.64) mL]，但差异无统计学意义（*P* > 0.05）（表 3）。

**3:** 跨叶静脉保留组与切断组的围术期资料和术后并发症情况 Perioperative data and postoperative complications in the translobar vein preservation and dissection groups

Items	Preserved group (*n*=12)	Severed group (*n*=36)	*P*
Gender			0.182
Male	8	20	
Female	4	16	
Age (Mean±SD, yr)	62.33±10.219	62.97±8.657	0.833
Surgical procedures			
RUL	9	34	
RLL/LLL	3	2	
Postoperative drainage days	2.33±1.92	2.44±1.90	0.862
Total drainage during 3 days after operation (mL)	604.92±229.64	705.42±265.02	0.247
Hospitalization days after operation	3.92±1.62	4.72±1.86	0.188
Complications			
Hemoptysis	0	5	0.312
Air leakage	1	7	0.659
Fever (≥38­ ℃)	3	6	0.617
Atelectasis	1	1	< 0.999
Pleural effusion	1	3	< 0.999
Hypoalbuminemia	2	11	0.574
LLL: left lower lob.				

## 3 讨论

研究^[11]^发现，肺静脉变异的存在会增加手术的并发症发生率，可能会导致术中意外出血、肺淤血、甚至严重的肺梗死。因此掌握肺静脉的跨叶现象对于安全地施行肺叶切除术至关重要。本研究首先将这些解剖位置特殊且对手术操作有直接影响的静脉总结为跨叶静脉，结合增强CT、3D-CTBA和手术录像对跨叶静脉的类型及频率进行系统性分析，并且发现跨叶静脉容易在手术中被忽视，对保留肺组织和术后恢复产生不利影响，同时本研究对跨叶静脉的处理策略做出相应的探讨。

肺跨叶静脉的发生率为82.26%，右肺远大于左肺（右侧80.65%；左侧11.94%），与Otsuji等^[5]^报道的数据略有差异（右侧56.5%；左侧13%），可能是因为本研究纳入了一部分异常汇入左心房或体循环静脉系统的肺静脉。在右肺水平裂和后斜裂部位，跨叶静脉发生率最高，Yamashita^[3]^将这种现象描述为V^4^、V^5^或V^6^汇入右肺上叶叶间侧静脉，发生率高达18.2%，尤其是在上、中叶融合在一起的时候。刘正津等^[4]^也发现，每一个肺裂融合的标本，都可见小静脉跨叶分布。本研究发现此类静脉多为上叶与中叶之间且走行于水平裂的叶间静脉V^3^b、汇入V^2^或V^3^的VX^4^或VX^6^，少数为右肺上叶前段的一些小静脉跨叶汇入V^5^。其次是右肺中间支气管和左肺下叶支气管后方区域，VX^2^或VX^1+2^常在此区域汇入IPV、LA或SPV，右侧较常见，文献报道的发生率为1.9%-5.7%^[12, 13]^，与本研究的结果一致，这类静脉容易在进行隆突下、肺门、叶间淋巴结清扫和打开斜裂后半部时被损伤，造成术中意外出血^[12]^。左肺SPV汇入IPV也比较常见，即上、下肺静脉共干，文献报道的发生率为8%-14%^[6]^，共干长度 > 1 cm的发生率为4%^[14]^，本研究统计的结果为4.84%，与既往研究基本一致。这种变异需要引起外科医生足够的警惕，一旦共干的左肺静脉被误断，若发现及时可以将断端与心房吻合^[15]^，若没有及时发现则只能行二次手术切除左肺上叶^[11]^。本研究还发现了一些新的变异：1例（0.3%）右肺VX^4^从基底段动脉外侧向后汇入V^6^，1例（0.32%）右肺VX^4^与VX^6^共干单独汇入左心房，有1例（0.32%）VX^4+5^与VX^6^、VX^7^a、VX^8^共干单独汇入左心房，在进行右肺下叶切除时应仔细游离、结扎相应分支，避免整支切断。另外，部分肺静脉异位引流是一种罕见的变异，文献报道^[16]^发生率约为0.2%，左上叶多见，它实际上属于从左向右的分流，在进行肺叶切除术时应慎重，手术可能会加重左向右分流，导致右心衰竭，必要时需行手术矫正异位引流。

通过回顾手术录像发现，75%汇入靶肺叶静脉的保留肺叶静脉会被切断，而这部分回流静脉被切断的肺组织在进行充气膨胀后停止通气，肺内气体在肺固有回缩力作用下迅速从气道内排出，随着肺萎陷小气道逐渐关闭，肺内残余气体无法被血流摄取，10 min后相应区域肺仍无法完全塌陷或塌陷缓慢^[17]^，与周围血运正常的、可以完全塌陷的肺组织之间形成清晰可见的膨胀-萎陷交界面，其原理类似于改良膨胀萎陷法^[18]^，间接反映这部分肺组织由于缺少回流静脉，血液循环功能受到影响。与之相反，术中保留跨叶静脉，则保留肺叶的功能不受影响，可以正常萎陷。另外，由于部分肺组织发生静脉性梗死，患者术后咯血、肺漏气的发生率可能会增加，这可能对术后肺功能恢复也有不利影响。因此，手术区域涉及到的跨叶静脉应尽可能保留。

跨叶静脉往往伴随着肺裂发育不完全^[5]^，它深藏在肺门深处，外科医生在术中很难发现这些跨叶静脉，因此术前通过CT图像与3D-CTBA去查看手术相关部位是否存在跨叶静脉就显得尤为重要^[19, 20]^。3D-CTBA有助于快速查看有无跨叶静脉，需要注意少数重建缺失和动静脉混淆的情况^[13, 21]^，而少数重建未显示的细小分支都可以在CT图像上观察到，所以这两个工具结合使用效果最好。而在手术过程中，除去部分分支过细的VX^6^很难保留，多数跨叶静脉可以通过调整手术方式得以保留，Aragaki^[22]^曾报道1例左肺下叶切除术中保留汇入下肺静脉的VX^1+2^，并强调术前使用3D-CTBA识别静脉变异的重要性。本文介绍了几种常见跨叶静脉的保留方式，例如汇入V^2^的VX^4^、汇入下肺静脉的VX^4^、叶间静脉V^3^b等，需要注意的是，术中发现叶裂发育不完全时要警惕跨叶静脉的存在，冒然切开肺裂容易造成出血、肺漏气或误断保留肺叶结构，此时可以采用无肺裂技术，也可以使用膨胀萎陷法帮助判断叶间裂的位置。

综上所述，肺静脉跨叶现象种类繁多，且部分类型发生率较高。然而大部分跨叶静脉在手术被忽视，这可能对手术安全和患者术后恢复产生不利影响，而术中保留跨叶静脉可能有助于减少并发症，加快术后恢复。因本研究样本量有限，并且为回顾性研究，研究结果尚需进一步证实。

本研究利用大量的胸部CT图像、三维重建和手术录像对肺部跨叶静脉的类型和发生频率进行了分析，并进一步结合临床实际，探讨跨叶静脉对肺叶切除术的影响，对于指导胸外科医生进行安全和精确的肺叶切除术具有一定的临床参考价值。
